# Role of the Gut Microbiota in the Increased Infant Body Mass Index Induced by Gestational Diabetes Mellitus

**DOI:** 10.1128/msystems.00465-22

**Published:** 2022-09-26

**Authors:** Qingyi Zhu, Xu Yang, Yingying Zhang, Chunjian Shan, Zhonghua Shi

**Affiliations:** a Women’s Hospital of Nanjing Medical University, Nanjing Maternity and Child Health Care Hospital, Nanjing, Jiangsu, China; b State Key Laboratory of Reproductive Medicine, School of Public Health, Nanjing Medical University, Nanjing, China; c Key Laboratory of Modern Toxicology of Ministry of Education, School of Public Health, Nanjing Medical University, Nanjing, China; Vanderbilt University Medical Center

**Keywords:** microbiota, gestational diabetes mellitus, 16S rRNA, BMI

## Abstract

The connection between gestational diabetes mellitus (GDM) and the offspring’s development, such as obesity, is well established. Emerging evidence indicates that the microbiota of the neonate's meconium is associated with maternal GDM status. To explore whether the association between GDM and infant body mass index (BMI) in early childhood is affected by the meconium microbiota, we recruited 120 mothers (60 healthy women and 60 with GDM) and their newborns from the Women’s Hospital of Nanjing Medical University. Meconium of 120 neonates was collected within a few hours after birth and sequenced using 16S rRNA sequencing analysis. Children’s BMI was measured at 12 months of age. The results revealed that infants born to mothers with GDM had increased BMI Z-scores at 12 months old and that the β-diversity of their meconium microbiota was reduced. Several genera were observed to be significantly different between the GDM and control groups. The genus *Burkholderia-Caballeronia-Paraburkholderia* and an untitled genus in the family *Enterobacteriaceae* enriched in neonates born to healthy mothers were found to be negatively associated with infant BMI by using regression analysis. A coabundance group depleted in the GDM group was correlated negatively with 12-month BMI and mediated 21.65% of the association between GDM and infant BMI by mediation analyses. This study provided evidence for the associations among maternal GDM, the meconium microbiota, and infant BMI. Maternal GDM was demonstrated to affect infant BMI, mediated by the gut microbiome. Gut microbiome interventions might represent a novel technique to decrease the risk of GDM-induced childhood obesity.

**IMPORTANCE** Using 16S rRNA sequencing analysis, regression analysis and mediation analysis were used to explore whether maternal gestational diabetes mellitus (GDM) changed the function and composition of the meconium microbiota and whether this explained the GDM-induced alterations of infant body mass index (BMI). This study showed that gut microbiome dysbiosis induced by maternal GDM might play an important role in the increased infant BMI during the first 12 months of life. Therefore, gut microbiome interventions might represent a novel technique to decrease the risk of GDM-induced childhood obesity.

## INTRODUCTION

Gestational diabetes mellitus (GDM), defined as the first recognized glucose intolerance during pregnancy, is a common complication of pregnancy (1). Pregnancy complicated with GDM suffers an increased risk of preeclampsia, preterm delivery, and the development of type 2 diabetes later in life (2). Offspring born to mothers with GDM are not only at an increased risk of immediate complications, such as macrosomia, shoulder dystocia, neonatal hypoglycemia, and respiratory distress (3), but also have an increased risk of developing long-term cardiovascular complications, metabolic syndrome, diabetes, and obesity (4, 5). Among these, childhood obesity has attracted increased public attention.

Several observational studies have indicated that offspring born to mothers with GDM have an increased risk of childhood obesity (5). In most studies, children born to mothers with GDM had an increased risk of overweight or obesity at 5 years or older (6, 7). Meanwhile, offspring born to mothers with GDM had higher body mass index (BMI) Z-scores in childhood (3 to 15 years) (8). Studies performed in children at 3 years or older revealed that environmental factors, such as exercise, lifestyle, or dietary habits, might have a major effect on childhood obesity (9, 10). After minimization of the impact of such environmental factors, a recent study in Turkey proposed that maternal GDM increased the risk of early childhood obesity in children aged 1 to 3 years old (11).

The underlying physiological mechanism of childhood obesity caused by maternal hyperglycemia remains unclear. According to a previous study, higher maternal glucose levels might decrease their offspring's insulin sensitivity and increase β-cell responsivity (12). Hyperleptinemia in women with GDM (13) might permanently reduce leptin sensitivity in the infant hypothalamus, which could have long-term effects on children’s energy balance and could be reflected in obesity (14).

The gut microbiota comprises a complicated collection of microorganisms that occupy the digestive tract of the host, which has important functions in host nutrition absorption, immunity, and metabolism (15). Dysbiosis of the gut microbiome might promote the development of GDM by regulating the host’s metabolism of various substances (16, 17). Interestingly, probiotics that might alter the composition of the gut microbiota (18) were used to treat mothers with GDM, resulting in a significant reduction in insulin resistance (19). In a newborn, the meconium is first colonized by the microbiota, derived mainly from the maternal gut, vagina, and skin (20), and codevelops with the host from birth (21). A mother's health status might affect her own microbiota during pregnancy, and the effects might be transmitted vertically to the offspring (22). GDM was found to be associated with the neonatal meconium microbiota and could change the diversity and composition of the neonate's gut microbiota (23). Adult obesity development is also highly affected by the gut microbiota (24). However, whether GDM-induced dysbiosis of the neonatal meconium microbiota is associated with children’s BMI remains unknown. Hence, our study aimed to explore whether maternal GDM changed the composition of the meconium microbiota and whether this explained the GDM-induced alterations of infant BMI.

## RESULTS

### Characteristics of study participants.

Participants’ characteristics are shown in Table 1. The mothers in the two groups had similar maternal ages, prepregnancy BMIs, parities, passive smoking statuses, and drinking statuses. Significantly higher fasting blood glucose and 1-h and 2-h post-oral glucose tolerance test (OGTT) glucose levels were observed in the mothers with GDM at 24 to 28 weeks of gestation than in those in the control group (*P* < 0.001). Moreover, the infant birth weight, BMI, and BMI Z-score at 12 months of age were higher in the GDM group than in the control group (*P* < 0.05).

**TABLE 1:** Characteristics of the study participants

Variable	Value for:		*P*
	GDM group (*n* = 60)	Control group (*n* = 60)	
Maternal^*a*^			
Age (mean ± SD, yr)	28.9 ± 3.66	28.5 ± 3.96	0.551
Prepregnancy BMI (mean ± SD, kg/m^2^)	21.9 ± 3.48	20.9 ± 2.62	0.099
OGTT_FBG (mean ± SD, mmol/L)	4.50 ± 0.70	4.06 ± 0.37	<0.001
OGTT_1 h (mean ± SD, mmol/L)	8.05 ± 2.09	6.54 ± 1.52	<0.001
OGTT_2 h (mean ± SD, mmol/L)	6.93 ± 1.58	5.87 ± 1.08	<0.001
Gestational age (mean ± SD, wk)	39.2 ± 1.27	39.2 ± 1.01	0.887
Parity [no. (%)]			1.000
Nulliparae	47 (78.3)	46 (76.7)	
Multiparae	13 (21.7)	14 (23.3)	
Passive smoking [no. (%)]			0.561
Never	42 (70.0)	38 (63.3)	
Ever	18 (30.0)	22 (36.7)	
Alcohol drinking [no. (%)]			0.439
Never	58 (96.7)	55 (91.7)	
Ever	2 (3.33)	5 (8.33)	
Neonatal			
Delivery mode [no. (%)]			1.000
Cesarean	44 (73.3)	44 (73.3)	
Vaginal	16 (26.7)	16 (26.7)	
Sex [no. (%)]			1.000
Boy	35 (58.3)	36 (60.0)	
Girl	25 (41.7)	24 (40.0)	
Birth wt (mean ± SD, g)	3,476 (400)	3,361 (360)	0.111
Breastfeeding [no. (%)]			0.777
Exclusive breastfeeding	8 (13.3)	6 (10.0)	
Mixed feeding	52 (86.7)	53 (88.3)	
Formula feeding	0 (0.00)	1 (1.67)	
Wt at 12 mo old (mean ± SD, kg)	10.4 (1.04)	10.1 (0.93)	0.048
Ht at 12 mo old (mean ± SD, cm)	76.6 (2.45)	76.8 (2.93)	0.782
BMI at 12 mo old (mean ± SD, kg/m^2^)	17.8 (1.24)	17.1 (1.27)	0.006
BMI Z-score at 12 mo old (mean ± SD)	0.77 (0.77)	0.31 (0.95)	0.004

a OGTT_FBG, OGTT fasting blood glucose value; OGTT_1 h, 1-h OGTT value; OGTT_2 h, 2-h OGTT value.

### Associations of GDM status, glucose levels, and BMI at 12 months of age.

**FIG 1:**
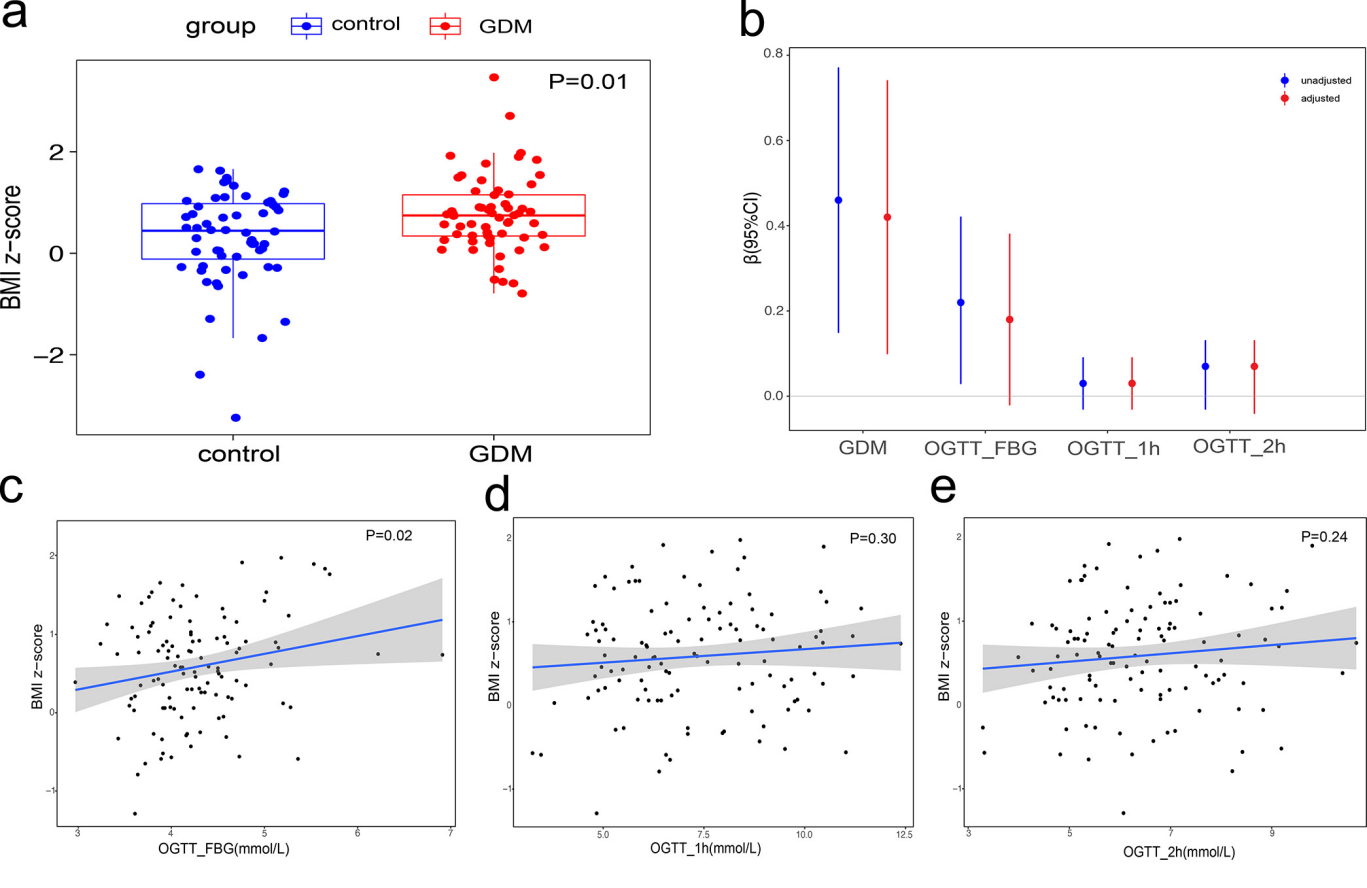
(a) Box plots showing BMI Z-scores of 12-month-old infants born to women with GDM and healthy controls. The central horizontal lines represent the medians, the top and bottom of the boxes are the 25th and 75th percentiles, and the points represent the BMI Z-scores of each sample. Maternal age, maternal prepregnancy BMI, and birth weight were adjusted as potential covariates. (b) Error bars showing the regression coefficients (β) of linear models of BMI Z-scores against GDM, fasting blood glucose (FBG) values, 1-h OGTT glucose values, and 2-h OGTT glucose values. The length of the bars indicates the 95% confidence interval. (b to e) Red indicates estimates adjusted for maternal prepregnancy BMI, maternal age, and birth weight, and blue indicates unadjusted linear models of BMI Z-scores at 12 months old against FBG values, 1-h OGTT glucose values, and 2-h OGTT glucose values.

In the GDM group, BMI Z-scores of infants at 12 months old were significantly increased (Fig. 1a). After adjustment for potential covariates, including maternal age, maternal prepregnancy BMI, and birth weight, GDM had the most significant influence on infant BMI Z-scores among the influencing factors mentioned above (*R*^2^ = 8.17%, *P* = 0.01). The offspring of the women with GDM had higher BMI Z-scores than the offspring of the control group. At the same time, it can be observed that infant BMI has an increasing trend with fasting blood glucose (unadjusted *P* = 0.02, adjusted *P* = 0.07), but no significant correlation was observed between infant BMI and 1-h or 2-h OGTT glucose values (Fig. 1c to e). Details of the linear models are shown in Fig. 1b and in Table S3 in the supplemental material.

10.1128/msystems.00465-22.6 TABLE S3 Details of the linear models testing the associations between GDM, glucose levels, and BMI at 1 year of age. Download Table S3, XLSX file, 0.01 MB. Copyright © 2022 Zhu et al. 2022 Zhu et al. https://creativecommons.org/licenses/by/4.0/ This content is distributed under the terms of the Creative Commons Attribution 4.0 International license.

### Associations between GDM status and the meconium microbiota.

**FIG 2:**
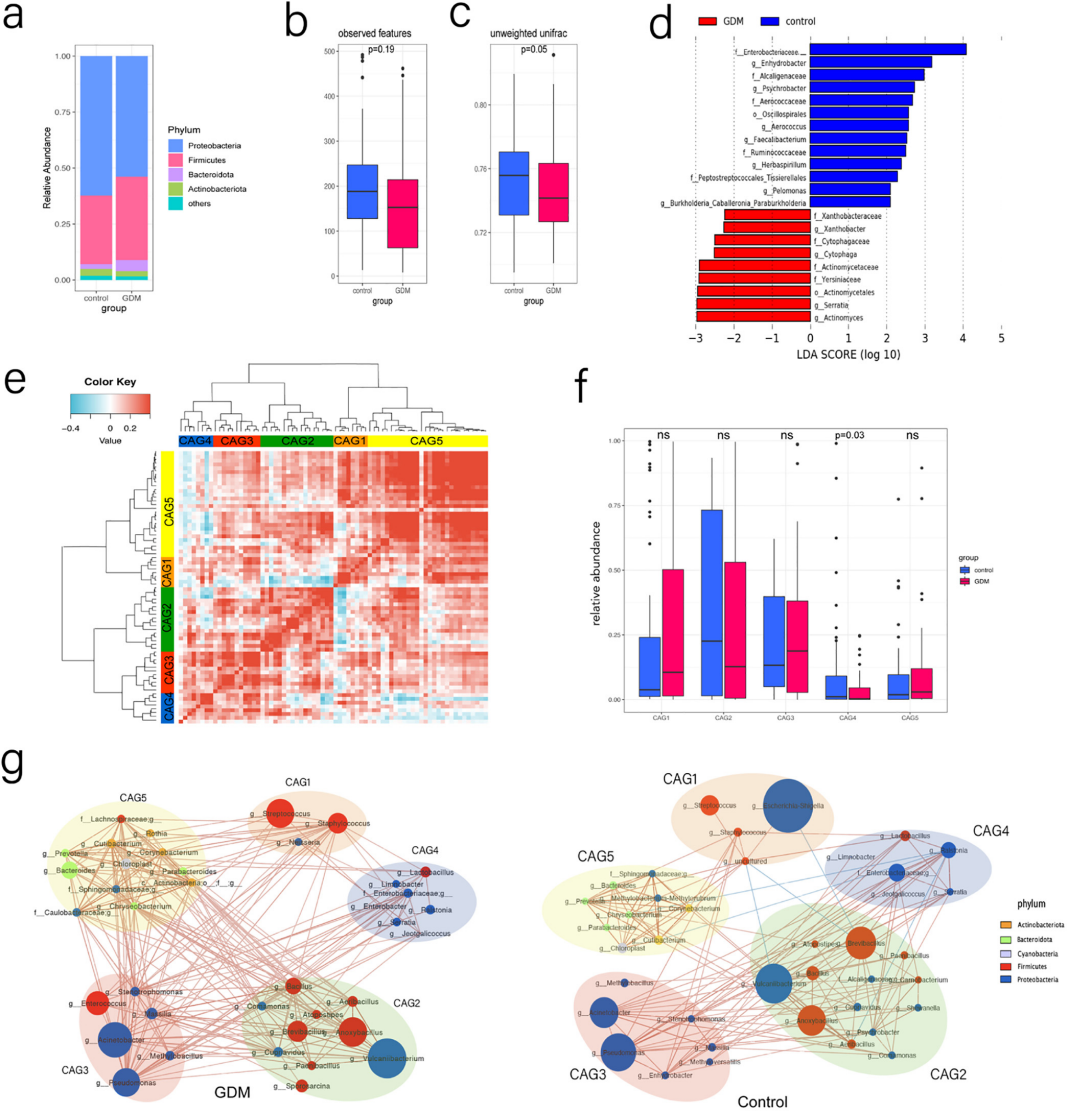
(a) Relative abundance of dominant phyla in the meconium microbiota in the GDM and control groups. (b) Indexes of α-diversity of the meconium microbiome in the GDM and control groups. (c) Indexes of β-diversity of the meconium microbiome in the GDM and control groups. (d) Difference dominant taxa were identified by LEfSe (LDA > 2.0) in the meconium microbiome between the two groups. (e) The top 72 most abundant genera were clustered into five groups according to hierarchical Ward clustering and the Spearman correlation coefficient. Kendall correlations between each genus are shown in the heat map. The most abundant genera in each CAG are as follows: CAG1 (genera Escherichia*-Shigella*, Streptococcus, and Staphylococcus), CAG2 (genera from *Bacillaceae* and *Brevibacillaceae*), CAG3 (genera within *Pseudomonadales*), CAG4 (genera from *Burkholderiaceae* and *Enterobacteriaceae*), CAG5 (*Bacteroides*). (f) The abundance of each CAG in the GDM and control groups. Differences were detected using nonparametric Wilcoxon tests. The abundance of CAG4 was depleted in the GDM group (*P* = 0.03). (g) Enrichment of the genera in the GDM and control groups is shown separately in two network diagrams. The sizes of the nodes represent the average relative abundance of each genus. The colors of the nodes represent the different phyla to which the genera belong. Spearman correlations between nodes are indicated by lines (Benjamini-Hochberg false discovery rate [FDR]-corrected *P* value < 0.01). Red lines indicate positive correlations; blue lines indicate negative correlations.

At the phylum level, *Actinobacteria*, *Bacteroidetes*, *Firmicutes*, and *Proteobacteria* represented the dominant taxa across all samples (Fig. 2a). The GDM group showed a relative decrease in the abundance of *Proteobacteria*. The observed features of the GDM group pointed to a decreasing trend in α-diversity compared with those of the control group (Fig. 2b). We compared the β-diversity indexes and found that infants born to women with GDM tended to have a lower β-diversity index than infants born to healthy mothers (Fig. 2c). Next, we used linear discriminant analysis effect size (LEfSe) to identify the key differential taxa between the two groups. In total, 12 genera, 8 families, and 2 orders were found to be significantly different between the two groups. At the genus level, we found that the abundances of *Enhydrobacter*, *Psychrobacter*, *Aerococcus*, *Faecalibacterium*, *Herbaspirillum*, *Pelomonas*, *Burkholderia-Caballeronia-Paraburkholderia*, and an untitled genus in the family *Enterobacteriaceae* were significantly lower and the abundances of *Xanthobacter*, *Cytophaga*, *Serratia*, and *Actinomyces* were significantly higher in meconium microbiota of infants born to mothers with GDM than in those born to healthy controls (Fig. 2d; Fig. S1 and Table S5).

10.1128/msystems.00465-22.1 FIG S1 Cladogram of dominant genera differing in the meconium microbiomes from the two groups. Download FIG S1, TIF file, 2.2 MB. Copyright © 2022 Zhu et al. 2022 Zhu et al. https://creativecommons.org/licenses/by/4.0/ This content is distributed under the terms of the Creative Commons Attribution 4.0 International license.

10.1128/msystems.00465-22.8 TABLE S5 LDA values and *P* values corresponding to Fig. 2d. Download Table S5, XLSX file, 0.01 MB. Copyright © 2022 Zhu et al. 2022 Zhu et al. https://creativecommons.org/licenses/by/4.0/ This content is distributed under the terms of the Creative Commons Attribution 4.0 International license.

To gain a more comprehensive understanding of the differences between the two groups in terms of their microbiota structures, the top 72 most abundant genera were classified into five coabundance groups (CAGs) according to the correlations between genera (Fig. 2e). The abundance of CAGs between the GDM and control groups was tested by nonparametric Wilcoxon tests. CAG4 was identified as a significantly differential CAG between the GDM and control groups (*P* = 0.03) (Fig. 2f). The relative abundance of the genera in each CAG is shown in Table S4. In the GDM group, CAG4 was reduced and mostly comprised genera from the *Burkholderiaceae* and *Enterobacteriaceae* families within the phylum *Proteobacteria* (Fig. S3). Then, we calculated the Spearman correlation for each genus in the different CAGs separately in the GDM and control groups. The internal correlations between the genera in the CAGs were different between the two groups (Fig. 2g).

10.1128/msystems.00465-22.3 FIG S3 Relative abundance of each genus in CAG4. Download FIG S3, TIF file, 0.7 MB. Copyright © 2022 Zhu et al. 2022 Zhu et al. https://creativecommons.org/licenses/by/4.0/ This content is distributed under the terms of the Creative Commons Attribution 4.0 International license.

10.1128/msystems.00465-22.7 TABLE S4 Relative abundance of genera in each CAG. Download Table S4, XLSX file, 0.1 MB. Copyright © 2022 Zhu et al. 2022 Zhu et al. https://creativecommons.org/licenses/by/4.0/ This content is distributed under the terms of the Creative Commons Attribution 4.0 International license.

### Relationships between BMI in infants at 12 months old and the meconium microbiota.

**FIG 3:**
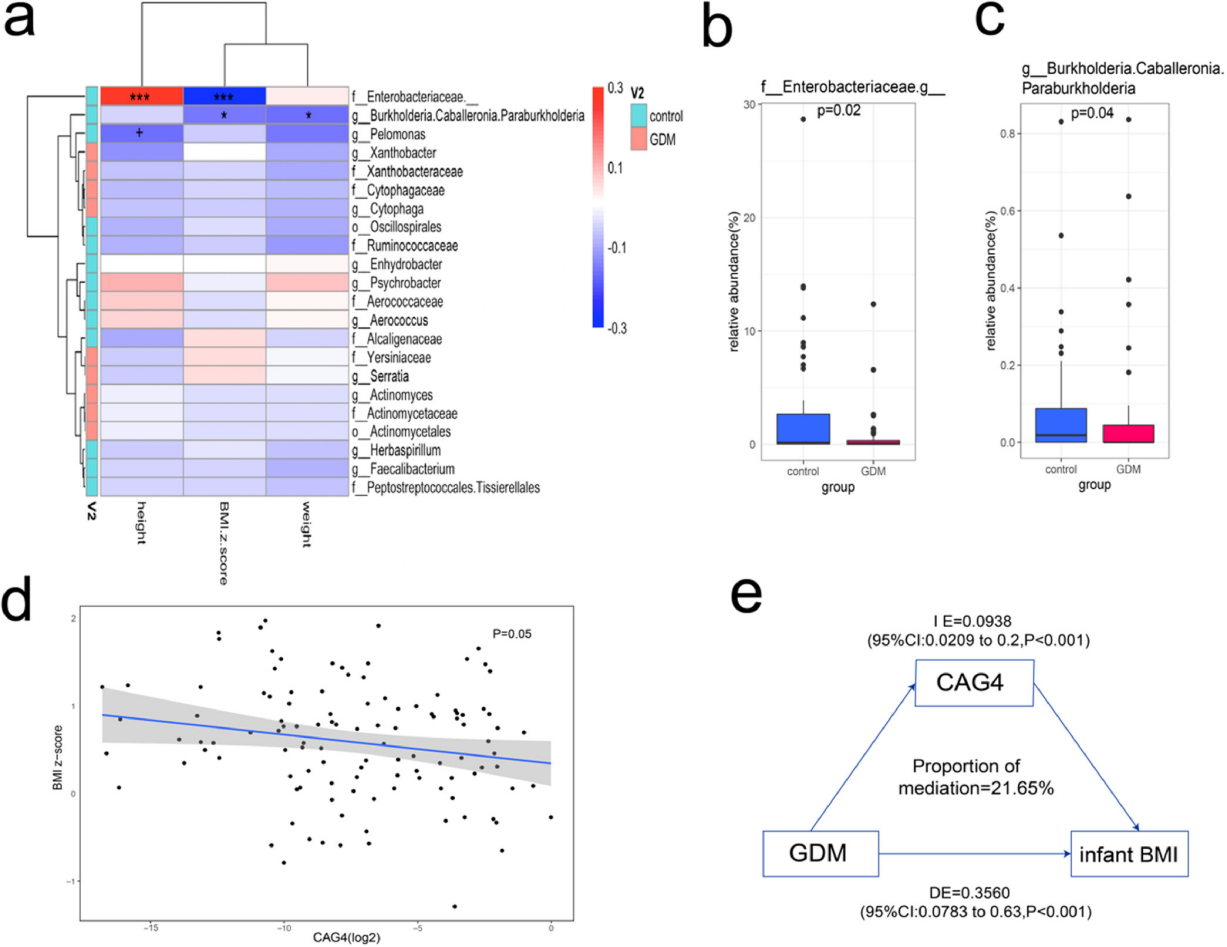
(a) Heat map of partial correlation coefficients between infant physical characteristics and the relative abundance of each dominant taxon that differed between the two groups. The statistical significance was calculated using a multivariate linear model (***, *P* < 0.001; *, *P* < 0.05; +, *P* < 0.1) Partial correlation coefficients and multivariate linear models were subjected to adjustment for birth weight, maternal prepregnancy BMI, and maternal age. (b and c) Relative abundance of the untitled genus in the family *Enterobacteriaceae* and the genus *Burkholderia-Caballeronia-Paraburkholderia.* These two genera correlated negatively with infant BMI at 12 months of age. (d) Linear model verifying the negative correlation between the relative abundance of CAG4 (log_2_ fold change) and infant BMI at 12 months of age (*P* = 0.05). (e) CAG4 mediated 21.65% of GDM-BMI association at 12 months of age (mediation effect of the β estimate = 0.2165, 95% CI, 0.05 to 0.57, *P* < 0.001). IE: indirect effect; DE: direct effect.

Lastly, we calculated the partial correlation coefficients between physical characteristics (height, weight, and BMI Z-score) of infants at 12 months old and the relative abundance of each key taxon that differed between the two groups. Multivariate linear models were used to test the significance of the correlations. Two genera enriched in the control group correlated negatively with BMI in infants at 12 months old (Fig. S2). Among these two genera, the untitled genus in the family *Enterobacteriaceae* correlated positively with infant height, and another genus, *Burkholderia-Caballeronia-Paraburkholderia*, in the family *Burkholderiaceae* correlated negatively with infant weight (Fig. 3a). The enrichments of these two genera in the control group were tested using nonparametric Wilcoxon tests and were identified to be statistically different (*P* = 0.02, *P* = 0.04) (Fig. 3b and c). A multivariate linear model was also used to verify the negative correlation between the relative abundance of CAG4 and infant BMI at 12 months old (*P* = 0.05) (Fig. 3d). Mediation analysis was then performed, which indicated that CAG4 mediated 21.65% of the GDM-BMI association in infants at 12 months old (mediation effect of the β-estimate = 0.2165, 95% confidence interval [CI], 0.05 to 0.57, *P* < 0.001) (Fig. 3e).

10.1128/msystems.00465-22.2 FIG S2 (a) Linear model verifying the negative correlation between the relative abundance of genus *Burkholderia-Caballeronia-Paraburkholderia* and infant BMI at 12 months of age (*P* = 0.03). (b) Linear model verifying the negative correlation between the relative abundance of the untitled genus in the family *Enterobacteriaceae* and infant BMI at 12 months of age (*P* = 0.04). Download FIG S2, TIF file, 0.8 MB. Copyright © 2022 Zhu et al. 2022 Zhu et al. https://creativecommons.org/licenses/by/4.0/ This content is distributed under the terms of the Creative Commons Attribution 4.0 International license.

## DISCUSSION

In the present study, associations between maternal GDM, the microbiota of meconium, and infant BMI were explored. Our results suggested that maternal GDM was associated with the increased infant BMI at 12 months of age, influenced by alterations of the meconium microbiota. Based on the above findings, gut microbiome interventions might become a novel technique to reduce the risk of GDM-induced childhood obesity.

According to a previous study (11), no significant association was found between maternal GDM and childhood obesity at 12 months of age but appeared from 2 years old and became more pronounced with age (8). Therefore, we decided to compare children's BMIs rather than obesity rates to assess the growth and development of the 1-year-old infants in this study. In addition to GDM, maternal obesity and high birth weight of children are also important factors affecting childhood obesity. Indeed, maternal obesity is considered an even more prominent risk factor than GDM (25) and might be a mediator between mother and child obesity (11). Thus, we adopted maternal prepregnancy BMI, maternal age, and neonate birth weight into our multivariate linear model and found that maternal GDM is an independent factor that increases infant BMI at 12 months of age. An increasing trend of infant BMI Z-scores with fasting blood glucose was also observed. These results were consistent with the development trend of GDM-induced childhood obesity in previous studies (5, 11, 26, 27) and revealed that the effects of GDM on child growth can be observed from a very early age.

The diversity of the microbiota was reportedly decreased in both stool samples from mothers with GDM and meconium samples from infants born to women with GDM (16, 17, 23). Herein, the α-diversity indexes were compared with the β-diversity indexes, which showed that infants born to women with GDM had lower unweighted UniFrac values than infants born to healthy mothers. Moreover, the α-diversity index (observed features) displayed a decreasing trend in the meconium microbiota of infants born to mothers with GDM. In addition, different numbers of genera were identified between the GDM and control groups. The number of genera belonging to the phylum *Proteobacteria* decreased the most in the GDM group, which was reportedly enriched in the meconium of infants born to nondiabetic mothers (23). We also classified the meconium microbiome into five CAGs, which correlated highly and might have similar functions. Among them, in the GDM group, CAG4, which mostly comprised genera from the families *Burkholderiaceae* and *Enterobacteriaceae*, decreased. Several species in the family *Burkholderiaceae* were reported to play major roles in the control of appetite and metabolism by encoding acyl coenzyme A (CoA) binding protein (ACBP) (28, 29). At the same time, the family *Enterobacteriaceae* was found to be enriched after diet intervention-induced BMI reduction (30). An untitled genus in the family *Enterobacteriaceae* was the most abundant genus in CAG4, and the function of the whole group might be similar to that of the dominant genus. Correlations between the genera in CAGs were found to be very different between the two groups, possibly verifying that maternal GDM affects the interactions between genera in the meconium microbiota.

In our study, various shifts in neonatal gut microbiota caused by maternal GDM correlated with infant BMI at 12 months of age. The genus *Burkholderia-Caballeronia-Paraburkholderia* and an untitled genus in the family *Enterobacteriaceae* correlated negatively with infant BMI at 12 months of age. The genus *Burkholderia-Caballeronia-Paraburkholderia* was reported to be associated with the bile acid cycle (31) and correlated with an individual's BMI in a study of cholangiocarcinoma (32). Besides, the coabundance group decreased in the GDM group and correlated negatively with infant BMI. The family *Burkholderiaceae*, one of the most abundant families in the coabundance group, was reported to be associated with BMI and insulin resistance mediated by the glutamate/glutamine ratio in plasma (33). The group also played an important role in mediating the association between GDM and infant BMI. These new findings revealed a potential functional role of the gut microbiota in GDM-induced increases in infant BMI. In previous studies, microbe-derived metabolites were proven to be involved in the process by which gut microbiota affects infant BMI (34). Also, offspring born to mothers with GDM may inherit their mothers’ high-energy-providing microbiota and alter their own carbohydrate and nucleotide metabolism (23). These results not only support our new findings but can help us explain the underlying mechanism by which maternal GDM affects offspring development mediated by gut microbiota. In addition to infant BMI, the untitled genus in the family *Enterobacteriaceae* was found to be associated with infant height at 1 year of age. However, there was no independent relationship between infant height and GDM, and we could not find any relevant studies to prove the existence of such a relationship. Therefore, the practical significance of the correlation between *Enterobacteriaceae* and offspring’s height remains to be further explored.

There are some strengths in our study. First, we combined maternal information with neonatal status at birth and long-term follow-up. This allowed us to explore potential transgeneration effects of GDM on childhood obesity. Second, we identified two protective genera and a coabundance group that are directly associated with infant BMI, which provided a theoretical foundation for further research on the use of probiotics for the early prevention and treatment of childhood obesity. However, there are several limitations to this study. The first one is that this study was a single-center study with a limited sample size. However, the participants included in this study were selected through a meticulous screening process, and we used the propensity score matching method to reduce the bias caused by confounding variables, which ensured the reliability and accuracy of the results. The second limitation is the resolution of 16S rRNA amplicon sequencing. It has been proposed in several studies that interactions between host and microbes generally occur in species- or subspecies-level variants (35, 36). Nonetheless, *16S rRNA amplicon sequencing* is technically mature and affordable for large-scale study. Based on our results, high-resolution shotgun metagenomic sequencing could be used to further identify the microbial species specific to infants born to mothers with GDM and to determine the interactions between gut microbes and childhood obesity, as well as to further explore the relationship between gut microbiome and infant BMI through functional profiling of microbial communities (37).

### Conclusions.

In the present study, we provided evidence for associations between maternal GDM, the meconium microbiota, and infant BMI. We found that gut microbiome dysbiosis induced by maternal GDM might play an important potential role in the increased infant BMI during the first 12 months of life. People are paying more attention to childhood obesity and related metabolic diseases; therefore, gut microbiome interventions might represent a novel technique to decrease the risk of GDM-induced childhood obesity. Further investigations using larger-sized samples and more precise sequencing methods are required to clarify the associations between GDM-induced obesity and the gut microbiome and the effectiveness and feasibility of intervening in the gut microbiota.

## MATERIALS AND METHODS

### Study design and participants.

We recruited the participants for this study from the Women’s Hospital of Nanjing Medical University (Nanjing, Jiangsu Province, China). Basic information regarding the participants, such as age, maternity history, family history of diabetes, and prepregnancy BMI, were collected using a demographic questionnaire. All participants were offered a standardized 75-g oral glucose tolerance test (OGTT) at gestational week 24 to 28. The criteria of the International Association of the Diabetes and Pregnancy Study Group (IADPSG) were used to diagnose GDM (38). A diagnosis of GDM must meet more than one of the following criteria: a 1-h OGTT glucose value greater than or equal to 10.0 mmol/L, a 2-h OGTT glucose value greater than or equal to 8.5 mmol/L, or a fasting blood glucose (FBG) value greater than or equal to 5.1 mmol/L. The hospital's electronic medical records were used to extract the following information: infant birth weight, mode of delivery, sex, and gestational age. Finally, the study included 60 women with GDM and 60 healthy controls who were matched using propensity score matching (ratio = 1:1) for their medical history, prepregnancy BMI, and maternal age. We excluded women reported being pregnant with twins or having chronic diseases requiring medication, substance or alcohol abuse, antibiotic usage within 3 months, preexisting diabetes, or a family history of diabetes. Detailed information is displayed in Table S1 in the supplemental material.

10.1128/msystems.00465-22.4 TABLE S1 Metadata information of the study population. Download Table S1, XLSX file, 0.02 MB. Copyright © 2022 Zhu et al. 2022 Zhu et al. https://creativecommons.org/licenses/by/4.0/ This content is distributed under the terms of the Creative Commons Attribution 4.0 International license.

All participants provided written informed consent for themselves and the neonates. The study was approved by the Ethics Committee of Nanjing Medical University (IRB no. [2016]009).

### Infant BMI.

When the children were 1 year old, they were subjected to routine physical measurements. Children’s length (to the nearest 0.1 cm using a stadiometer) and weight (to the nearest 0.1 kg using an electronic scale) were measured. The World Health Organization reference (39) was used to calculate sex- and age-specific BMI Z-scores. The formula for Z-score is shown as Z = (x − μ)/σ, (where x is infant BMI, μ is the sex- and age-specific population mean, and σ is the sex- and age-specific population standard deviation [SD]) (40).

### Sample collection, DNA extraction, and 16S rRNA sequencing.

We collected the first-pass meconium samples (approximately 200 mg) from 120 infants born to the participants on sterilized diapers within a few hours after birth and stored the samples at −80°C until DNA extraction. Genomic DNA was extracted using a QIAamp fast DNA stool minikit (Qiagen, Hilden, Germany) in a sterile environment. The 16S rRNA V3 hypervariable region was PCR amplified using primers 338F (5′-ACTCCTACGGGAGGCAGCAG-3′) and 806R (5′-GGACTACHVGGGTWTCTAAT-3′). Gel electrophoresis was used to check the amplicons, which were purified using an Agencourt AMPure XP kit (Beckman Coulter, Brea, CA, USA). A Qubit 2.0 fluorometer (Thermo Scientific, Waltham, MA, USA) and an Agilent Bioanalyzer 2100 system (Santa Clara, CAS, USA) were used to perform quality testing on the amplification products constituting the 16S V3 library. Finally, the Illumina MiSeq platform (San Diego, CA, USA) was used to sequence the library to generate 2 × 250 bp paired-end reads.

### Sequencing data processing.

Quality control and follow-up analyses of the raw sequencing reads of the 16S rRNA genes were conducted using QIIME2 V.2021.8 (41). The 120 samples generated 10,545,284 of 16S rRNA clean reads (mean reads per sample = 85,155; SD = 43,761). Using the DADA2 pipeline (version 1.6) (42), these sequences were identified as amplicon sequence variants (ASVs). The SILVA database (version 138.1) was used to annotate the taxonomic information (Table S2) (43). The QIIME2 pipeline was used to calculate the α-diversity metrics (Shannon index, Simpson index, observed features, and chao1 index) and the β-diversity index (unweighted UniFrac distance). The lowest sequence depth to which all samples were rarefied was 37,402. Genera with a median proportional abundance of <0.01% across all samples were removed.

10.1128/msystems.00465-22.5 TABLE S2 Bacterial composition in meconium samples. Download Table S2, XLSX file, 0.5 MB. Copyright © 2022 Zhu et al. 2022 Zhu et al. https://creativecommons.org/licenses/by/4.0/ This content is distributed under the terms of the Creative Commons Attribution 4.0 International license.

### Microbiome CAG network.

After removal of the genera whose sum of relative abundances was less than 0.1, coabundance groups (CAGs) were constructed for the remaining top 72 most abundant genera. These genera had a total abundance of 97.5%. The Kendall correlation-defined coabundant groups were visualized as a Spearman correlation distance metric by hierarchical Ward clustering in the Made4 package (44). The adonis function in the vegan package (45) was used to determine the number of clusters, based on the number of significant differences in the pairwise adonis test scores among the original Kendall correlation-defined groups. The expression level of each CAG was specified as the sum of relative abundances of the genera classified to this CAG. The abundance of CAGs between the GDM and control groups was tested by nonparametric Wilcoxon tests. Cytoscape 3.7.1 (46) was used to separately visualize the Spearman correlation of the genera in the different CAGs in the GDM group and control group (Data Set S1).

10.1128/msystems.00465-22.9 DATA SET S1 Codes used in this study. Download Data Set S1, TXT file, 0.00 MB. Copyright © 2022 Zhu et al. 2022 Zhu et al. https://creativecommons.org/licenses/by/4.0/ This content is distributed under the terms of the Creative Commons Attribution 4.0 International license.

### Statistical analysis.

To describe the basic characteristics of the participants, the mean ± SD was used to show continuous variables and numbers (percentages) were used to show categorical variables. Student's *t* test and chi-square tests were used to compare the differences between the GDM group and the control group. Multivariate linear models were used to test the associations of GDM, glucose levels, and BMI at 12 months of age and the associations between meconium microbiota relative abundance and infant physical characteristics at 12 months of age. To exclude the influence, potential covariates were included in the adjusted models. Outliers were excluded using the boxplot.stats function in the grDevices package in the models shown in Fig. 1c to e and Fig. 3d. The calculation formulas of Tukey's box plot method (1.5 times the interquartile range [IQR]) (47) in this function are shown as follows: lower limit = P25 − (1.5 × IQR); upper limit = P75 + (1.5 × IQR). (P25: 25th percentile; P75: 75th percentile) Ultimately, 6 samples were removed, and the remaining 114 samples were used to build the models. Differences in α-diversity, β-diversity, and the relative abundance of specific bacterial genera between the two groups were tested using nonparametric Wilcoxon tests. The linear discriminant analysis (LDA) effect size (LEfSE) (logarithmic LDA scores > 2.0) (48) was used to identify the key bacterial taxa that differed between the two groups at the genus level. Partial correlation coefficients were calculated using the pcor function in the ggm package (49). To determine whether the meconium microbiota exerted a mediation function in the association between GDM and the offspring’s BMI at 1 year old, mediation analysis was carried out using the mediation package in R (50). All statistical analyses were conducted in R v4.1.1 (51) (Data Set S1).

### Data availability.

All sequence data have been deposited in NCBI under the accession numbers shown in Data Set S2b.

10.1128/msystems.00465-22.10 DATA SET S2 (a) Data used in the codes. (b) Accessions of sequence files in NCBI. Download Data Set S2, XLSX file, 0.07 MB. Copyright © 2022 Zhu et al. 2022 Zhu et al. https://creativecommons.org/licenses/by/4.0/ This content is distributed under the terms of the Creative Commons Attribution 4.0 International license.
