# Supplementary figures and images for: Role of the Gut Microbiota in the Increased Infant Body Mass Index Induced by Gestational Diabetes Mellitus
Source: mSystems. 2022 Sep 26;7(5):e00465-22. doi: 10.1128/msystems.00465-22 (PMC9601173; doi:10.1128/msystems.00465-22)

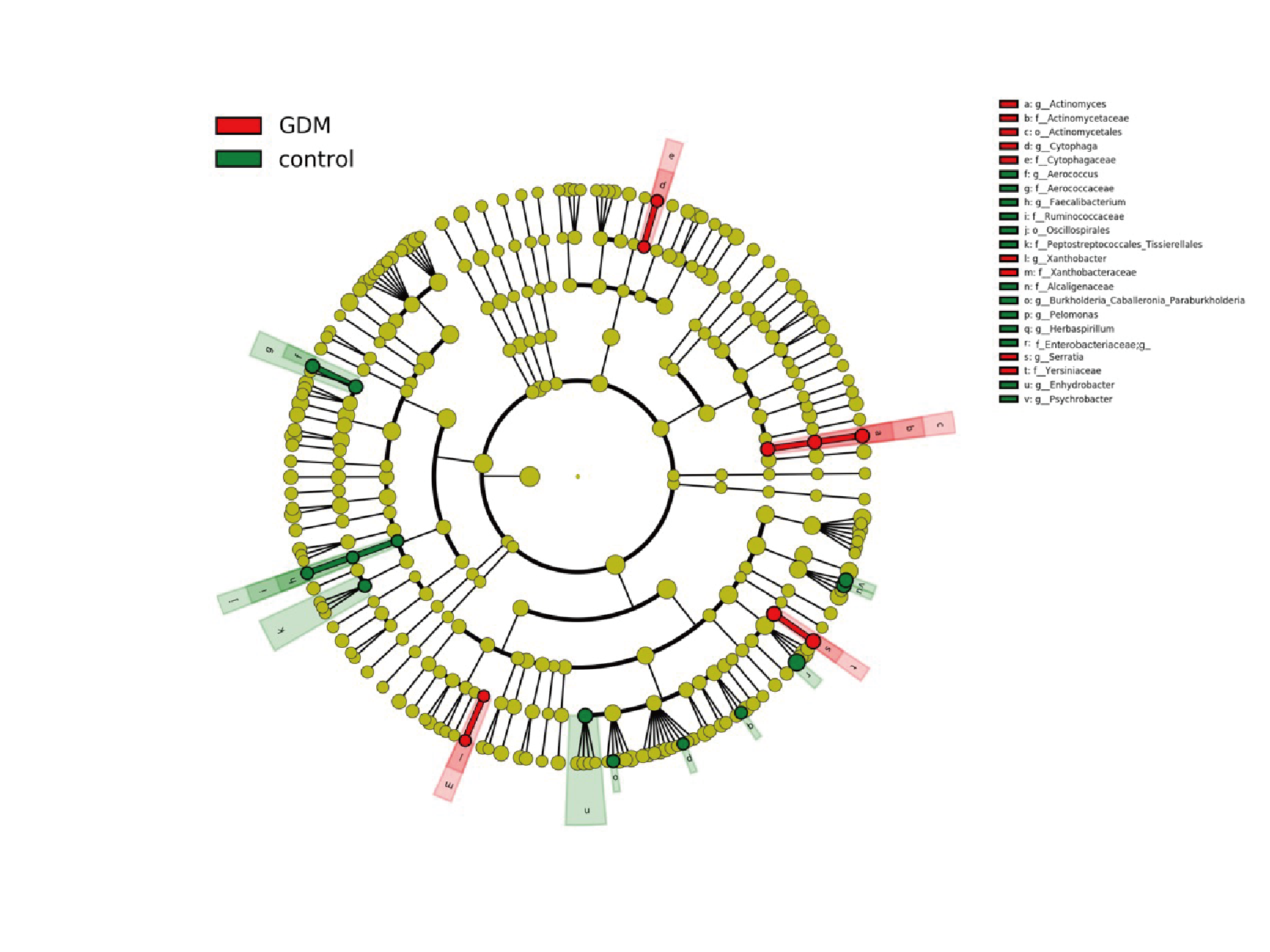

Supplement: FIG S1 [file msystems.00465-22-s0001.tif]

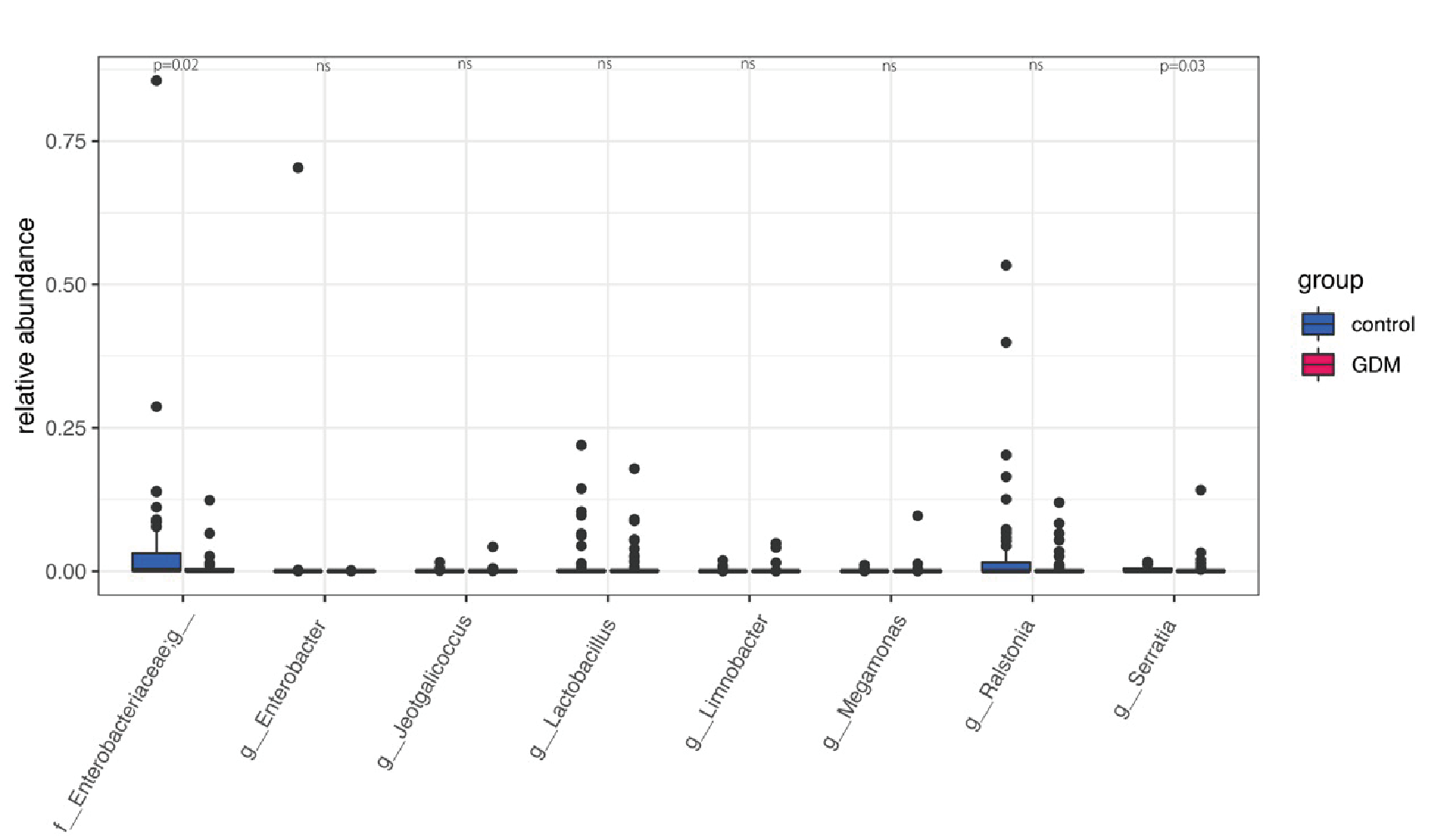

Supplement: FIG S3 [file msystems.00465-22-s0003.tif]

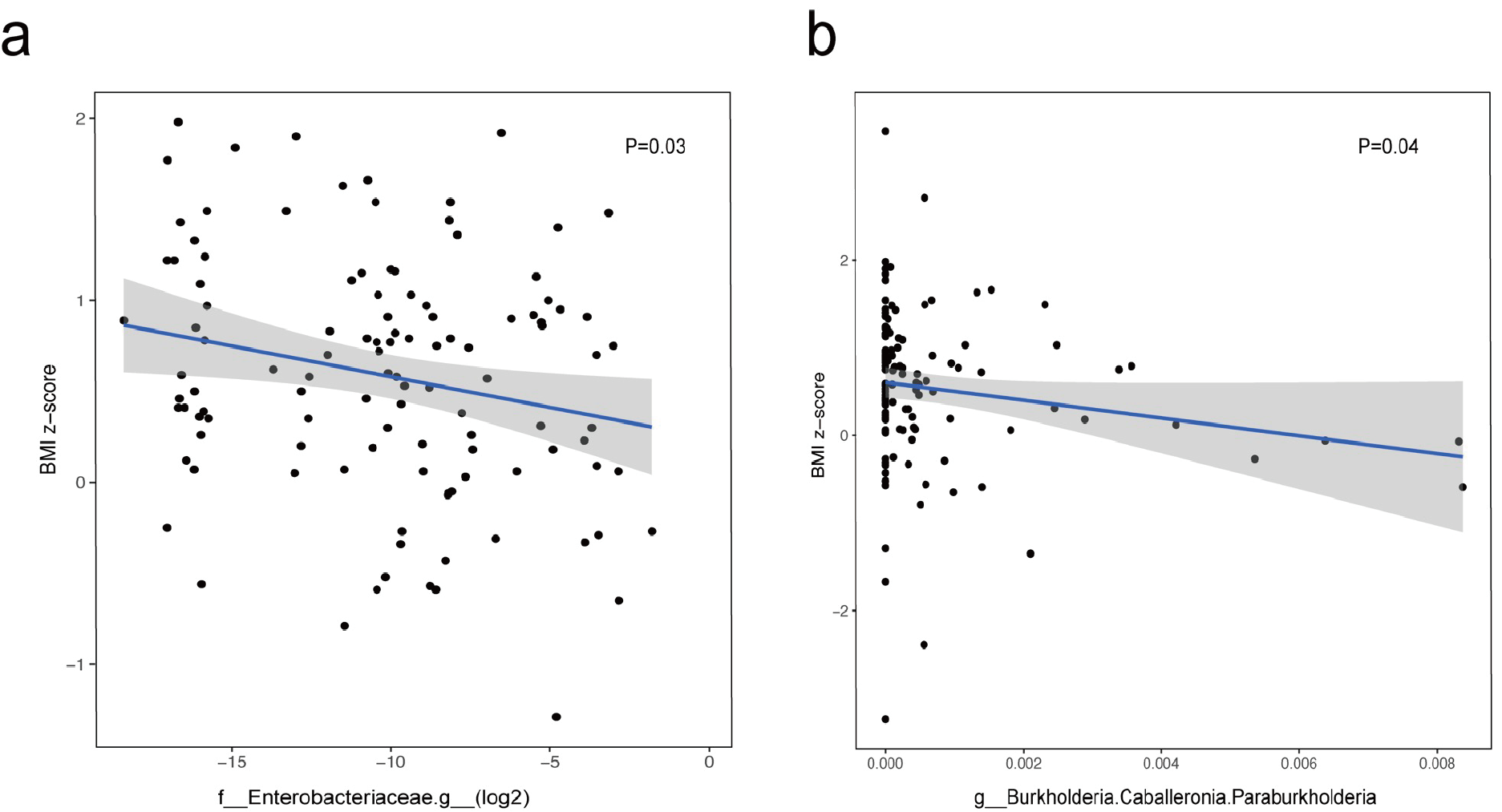

Supplement: FIG S2 [file msystems.00465-22-s0002.tif]
